# Antimicrobial susceptibility of *Escherichia coli*,* Klebsiella pneumoniae*, and *Enterococcus species* and the associated risk factors in poultry farms in Blantyre City: a wake-up call to the one health approach

**DOI:** 10.1186/s12917-025-05189-7

**Published:** 2025-12-23

**Authors:** Muonaouza Deleza, Petros Chigwechokha, Peter Mwale, Lecollins Mthirakuwiri, Williams Mwantoma, Brighton Nkunika, Samson Batani, Chisomo Redson, John Njalam’mano, John Taulo, Wilfred Kadewa, Alfred Maluwa, Henson Kainga

**Affiliations:** 1 Department of Energy Resources, Ndata School of Climate and Earth Sciences, Malawi University of Science and Technology, P.O. Box 5196, Limbe, Malawi; 2 Department of Biological Sciences, Academy of Medical Sciences, University of Science and Technology, P.O. Box 5196, Limbe, Malawi; 3 Department of Animal Health and Livestock Development, Central Veterinary Laboratory, P.O. Box 527, Lilongwe, Malawi; 4 Blantyre Regional Veterinary Laboratory, P.O. Box 96, Blantyre, Malawi; 5 Blantyre Society for the Protection and Care for Animals, P.O. Box 124, Blantyre, Malawi; 6 Department of Management and Leadership Studies, University of Malawi, P.O. Box 280, Zomba, Malawi; 7 Department of Water Resources, Ndata School of Climate and Earth Sciences, Malawi University of Science and Technology, P.O. Box 5196, Limbe, Malawi; 8 Directorate of Malawi Environmental Protection Authority, P/Bag 317, Lilongwe 3, Malawi; 9 Directorate of Research and Outreach, Malawi University of Science and Technology, P.O. Box 5109, Limbe, Malawi; 10 Department of Veterinary Epidemiology and Public Health, Faculty of Veterinary Medicine, Lilongwe University of Agriculture and Natural Resources, P.O. Box 219, Lilongwe, Malawi

**Keywords:** Antimicrobial resistance, Broilers, Layers, Poultry, Public health, Risk factors, One health

## Abstract

**Background:**

Small scale poultry farming often involves intensive farming which includes the extensive use of antibiotics for growth promotion and disease prevention. This can be used as a focal point for understanding the spread of resistant bacteria among the livestock, human and the environment interface. This study aimed at unravelling the antimicrobial use and resistance in small scale poultry farmers in Blantyre, Malawi.

**Methods:**

Samples from chickens, humans and the environment were collected from various poultry farms to assess resistant profiles for *Escherichia coli*,* Klebsiella pneumoniae*, and *Enterococcus* species. Bacterial culturing and disc diffusion methods were used to isolate and assess the antimicrobial resistance (AMR) profiles of the isolated bacteria respectively. Logistic and linear regression tests were used to assess potential risk factors for the AMR.

**Results:**

The study revealed uniform resistance profiles of *E. coli* to ampicillin, cefotaxime, ciprofloxacin, gentamicin and sulfamethoxazole trimethoprim across samples from humans, chickens and their shared environment. The *K. pneumoniae* resistance profiles to sulfamethoxazole trimethoprim were higher in chicken and environmental samples compared to the human samples. The resistance profiles for *Enterococcus* species to vancomycin, linezolid, ciprofloxacin and tigecycline were similar across the three sample types. Nevertheless, *E. coli* and *K. pneumoniae* showed 100% sulfamethoxazole trimethoprim, while the *Enterococcus species* showed 80% resistance to vancomycin from all the three sample types. Farms that did not observe withdrawal periods were five times more likely to experience AMR (OR = 5.32, 95% CI: 1.89–14.94, *p* = 0.013) than the farms that observed withdrawal periods. Farms that had inadequate waste management practices were three times more likely to experience AMR (OR = 3.42, 95% CI: 1.22–9.59, *p* = 0.037) than the farms that had adequate waste management practices.

**Conclusions:**

The study confirms occurrence of AMR in the poultry farming, humans and the environment influenced by both biological and management factors. The similar resistance patterns across three sample types suggest a dynamic transmission of resistant bacteria at the human-animal-environment interface, hence emphasizing the need for a One health approach to mitigate AMR.

**Supplementary Information:**

The online version contains supplementary material available at 10.1186/s12917-025-05189-7.

## Introduction

According to World Health Organization (WHO), the common infections across the globe could become untreatable due to increasing AMR, potentially leading to a post-antibiotic era [1]. Antimicrobial resistance has become a silent pandemic, undermining the effectiveness of antibiotics and other antimicrobial agents [1, 2]. One of the major drivers of AMR is the misuse and overuse of antibiotics in both human healthcare and agriculture, with the livestock industry playing a crucial role, particularly through use of antibiotics in feed as growth promoters and for prophylactic treatment [3].

Pathogens such as *Escherichia coli (E. coli)*,* Klebsiella pneumoniae (K. pneumoniae)*, and *Enterococcus* species are among the most commonly isolated pathogens in poultry farms [4]. These bacteria can cause a variety of infections, including gastrointestinal diseases in humans and respiratory infections in poultry [5]. The development of resistance in these bacterial species is of particular concern due to their ability to acquire and transmit resistance genes through plasmids and other mobile genetic elements [5]. This not only exacerbates the spread of resistance within poultry farms but also increases the risk of transferring these resistant strains to humans, such as farm workers and consumers of poultry products [6].

Resistant bacteria can spread from poultry to humans through several routes, including direct contact with animals, consumption of contaminated meat, and exposure to contaminated environments [7]. Farm workers are at risk of exposure, as they regularly handle poultry and are involved in farm operations with potential to aerosolize bacteria, such as cleaning poultry houses, handling crates and egg trays. Poultry house dust and feces serve as important reservoirs for resistant bacteria, which persist in the environment and be transmitted to other farms or surrounding communities [8].

In Malawi, poultry farming plays a crucial role in food security and economic development, especially in urban areas such as Blantyre city, where rising demand for poultry products has led to the rapid expansion of commercial farms [9]. However, this growth has been accompanied by inadequate regulation and monitoring of antimicrobial use (AMU), particularly in controlling bacterial infections like those caused by *E. coli*,* K. pneumoniae*, and *Enterococcus* species [10]. Farmers often administer antibiotics without veterinary supervision or knowledge of the potential risks associated with improper use [11]. Consequently, this unchecked use of antibiotics has led to the development and spread of AMR, threatening not only the health of poultry but also the livelihoods of farmers and public health in general. Poultry is suspected to generate AMR pathogens that increase the AMR burden on human health, livestock production, and the cost of living for communities and the nation [12].

Although AMR burden has been documented in human, animal, and environmental sectors in Malawi, existing studies remain fragmented [13–17]. For instance, Chisembe et al. provided the national AMR burden of *E. coli* in broiler chickens in Malawi with 95% of the *E. coli* isolates being classified as multidrug-resistant (MDR) [13]. Kalumbi et al. also highlighted the prevalence of AMR in livestock in Bvumbwe, Thyolo district where *E. coli* in chickens showed resistance to gentamicin [17]. In addition, Chatatanga presented AMR baseline data from both human (from hospital set-up) and animal health (from poultry national active surveillance) that indicated 100% resistance of *E. coli*. to ampicillin, 35% resistance to ciprofloxacin and 90% resistance to sulfamethoxazole trimethoprim in poultry processed at Blantyre regional veterinary laboratory [16]. These studies provide critical insights into AMR burden, however lacks a One Health approach, particularly in assessing environmental reservoirs such as surfaces in livestock pens.

Owing to One Health nature of AMR burden; where human, animal, and environmental health are interconnected, it is essential to understand the prevalence of resistant bacteria across three sample types (human, animal, and environmental) within the poultry farming context. Identifying risk factors associated with the emergence and spread of resistant strains in poultry farms is fundamental to mitigate AMR at source [18]. AMR in Malawi’s poultry farms is a growing concern that requires urgent attention, particularly in urban areas like Blantyre and Lilongwe, where commercial poultry farming is concentrated. The study sought to establish the prevalence and AMR patterns of *E. coli*,* K. pneumoniae*, and *Enterococcus* species across human, animal, and environmental samples in poultry farms. Further, the study identified potential risk factors contributing to the spread of resistant bacteria at human-livestock- environment interface. This information is critical for guiding future policy decisions, shaping national AMR surveillance efforts, and informing farm-level interventions aimed at reducing the use of antibiotics and minimizing the spread of resistant bacteria.

## Materials and methods

### Study design, study area and study units

A cross-sectional study design was used to conduct this research. The samples were collected from randomly selected commercial poultry farms in urban and peri-urban areas of Blantyre city. The farm locations were randomly selected from the list of Extension Planning Areas (EPAs) as outlined in the register book available at the Blantyre District Agriculture Office (BDAO). The Blantyre small and medium scale poultry association verified 180 poultry farms from the BDAO register book.

**Fig. 1:**
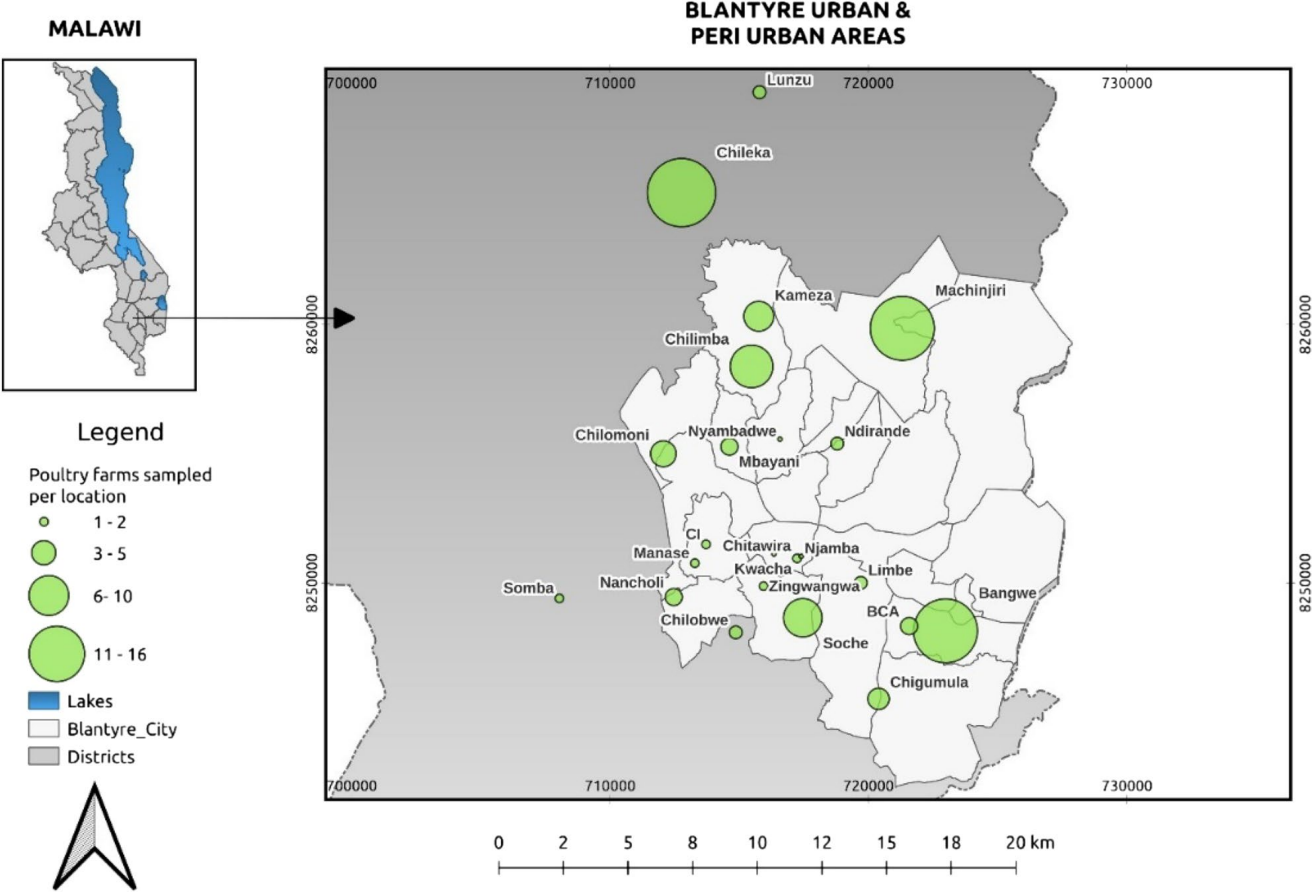
Location of commercial poultry farmers where data was collected

Sample size was estimated using the online Raosoft sample size calculator (www.raosoft.com/samplesize/html) based on following assumptions: 5% margin of error, 95% confidence interval and 50% response distribution. Using Microsoft™ Excel Spreadsheet (Microsoft Office Excel^®^2019). A total of 123 poultry farmers were randomly recruited into the study. These farmers were proportionally distributed across Extension Planning Areas (EPAs) (Fig. 1). All the 123 poultry farmers were then interviewed to assess risks factors associated with occurrence of AMR. From these 123 poultry farms, 11 farms (7 broiler and 4 layer farms) were then purposively recruited for sampling for further laboratory analysis based on the following inclusion criteria; For broilers, those that were about to enter the food chain (5 weeks old and above). For the layers, were those that were laying eggs or spent layers (18 weeks and above). Also, only those with healthy chickens were included for sampling. The health status was determined by the history and observing the chickens from a distance. Table 1 describes how the healthy chickens were determined. The farms that had sick chickens were excluded for sample collection but the farm owners or managers were still eligible to be interviewed.

**Table 1:** Determination criteria for healthy chickens

Feature	Description
General appearance	A healthy chicken will appear alert and avoid strangers if it is in a well-lit area.
Eyes	Healthy chicken eyes are clear and shiny.
Nostrils	Both nostrils of a healthy chicken should be clear and open, with no discharge.
Mouth	A healthy chicken should breathe with the mouth closed, except in very hot conditions.
Wings	The wings of healthy chickens should be carried close to the body. The wings should not droop or look twisted.
Feathers	The feathers of healthy chickens should be smooth and close to the body.
Feet and toes	You should not see any limping. Importantly, it is normal for broilers chickens to waddle, and this should not be mistaken with limping.
Vent	The feathers under the tail of a healthy chicken around the vent (the common opening for feces, mating, and passing eggs) should not be matted with feces.

From each farm, three types of samples were collected. These included one hand swab from the farmworker in close contact with the chickens, one pooled fecal sample per chicken house (consisting of four samples taken from different quadrants of each chicken pen) and one pooled environmental sample per chicken house (also consisting of samples from different quadrants of the chicken pen). A maximum of 10 samples were collected from each poultry farm. This yielded a total of 71 samples (40 samples from broiler farms and 31 samples from layers). The study considered only broiler and layer poultry farms located within Blantyre city.

### Questionnaire design and administration

A semi-structured questionnaire was adapted from the previous study conducted in Zambia [19]. KoboCollect software v2021.2.4 was used (Supplementary information I) to administer the interviews. These interviews were done in English and Chichewa language based on the language preference by the farmers. The questionnaire was pre-tested for objectivity, relevance, accuracy, clarity, simplicity, and understandability by a team of experts at Malawi University of Science and Technology (MUST), Lilongwe University of Agriculture and Natural Resources (LUANAR), Department of Animal Health and Livestock Development (DAHLD), Blantyre regional veterinary laboratory (BRVL), BDAO and Central Veterinary Laboratory (CVL) [20]. The questionnaire captured information on farm characteristics, AMU, biosecurity measures, waste management practices, and knowledge of AMR and One health, withdrawal period, hygiene practices.

Farmers were interviewed by the principal investigator or 2 trained enumerators. Observational checks were also done during the interview to verify self-reported practices such as antibiotics used. The questionnaire was piloted to 10 poultry farmers that had no poultry during the period of data collection in October 2024 and the data was excluded during the analysis. Data was collected in the period from October to December 2024 because this is the period when most farmers keep chickens to meet the demand for poultry meat and eggs that surges during the Christmas and New year festivals.

### Sample collection from poultry farms

Aseptic techniques were employed before entering each poultry farm such as cleaning gumboots with Virkon ^TM^ disinfectant (Thermo Fischer Scientific, USA) wearing gloves, headgear, lab coats and other personal protective equipment (PPE) according to Zakariya et al. [21]. A set of samples was collected from each sampled poultry farm such as hand swabs from farm workers, chicken fecal samples, and dust from the poultry houses.

Prior to poultry sample collection, each chicken house was divided into four quadrants. A pooled sample of fresh chicken droppings was collected using a sterile metal spatula. The sample was transferred into a sterile Ziplock bag (Thermo Fisher Scientific, USA) according to Amosun et al. [22]. Pre-moistened sterile swabs with buffered peptone water were used to collect samples from the hands of farm workers by rubbing the Sterile Culture swabs (BD BBL™ CultureSwab) across their hand surfaces. Dust from chicken houses was also collected using Sterile Culture Swabs pre-moistened with buffered peptone water. The swabs were rubbed over the surface walls using a zigzag motion to ensure contact with entire surface. All the samples were then labelled and transported in cooler boxes with ice packs at 4 °C to the laboratory within 4 h of sample collection.

### Bacterial isolation and identification

All sample types were enriched with Buffered Peptone Water (BPW). For the fecal samples, 10 g of poultry fecal sample was added to 90 mL of BPW. For the poultry dust swabs, four swabs were submerged in 50 mL of BPW to make one pooled sample. For the hand swabs, each swab was submerged in 10 mL of BPW. These samples were then incubated for 24 h at 37 °C. Following the incubation, 0.01 mL of each sample was inoculated onto MacConkey Agar for the isolation of *E. coli* and *K. pneumoniae*, and onto Enterococcus Agar for the isolation *Enterococcus* species, as described by Amosun et al. [22]. On MacConkey, presumptive *E. coli* colonies were small, pink, round, flat colonies while those of *K. pneumoniae* were, large, pink, round and mucoid. Typically, small pink or dark red flat colonies on Enterococcus Agar were considered presumptive for *Enterococcus* species. A maximum of three colonies on each plate with presumptive colonies were sub cultured on Blood Agar for 24 h at 37 °C to obtain pure colonies which were further identified using Matrix-Assisted Laser Desorption Ionization-Time of Flight Mass Spectrometry (MALDI-TOF ^®^ MS, bioMerieux) machine. Bacterial identification on the MALDI -TOF was done following Seng et al. [23]. The confidence value of greater than or equal to 99.9% was considered high confidence for species identification. This interpretation of the results was based on the manufacturer’s guidelines. The results of confidence value of below 99.9% were repeated and if below this threshold, they were reported at genus level. All the culture swabs and dehydrated media used were from Dico laboratories, Becton, Dickinson and Company, USA.

### Antimicrobial susceptibility testing of isolated bacteria

Immediately after bacterial isolation and identification, antimicrobial susceptibility testing (AST) was performed using the disc diffusion method to evaluate the resistance patterns of *E. coli*,* K. pneumoniae*, and *Enterococcus* species to commonly used antibiotics according to Mwansa et al. [24]. Antibiotics were selected according to the European Committee on Antimicrobial Susceptibility Testing (EUCAST) 2024 guidelines [25] in consideration to those recorded to be used in the poultry farms from interviews with poultry farmers. The antibiotic disks used and Mueller-Hinton Agar were manufactured by Dico laboratories, Becton, Dickinson and Company, USA. The concentrations the antibiotic disks were as follows: for *E. coli*; ampicillin 10 µg, cefotaxime 5 µg, ciprofloxacin 5 µg, gentamicin 10 µg, sulfamethoxazole-trimethoprim 1.25 µg, for *K. pneumoniae;* cefotaxime 5 µg, ciprofloxacin 5 µg, gentamicin 10 µg, sulfamethoxazole-trimethoprim 1.25 µg, and for *Enterococcus* species: ampicillin 2 µg, ciprofloxacin 5 µg, linezolid 10 µg, vancomycin 5 µg, and tigecycline 15 µg. To ensure quality of the antibiotics disks is controlled, *E. coli* ATCC 25,922 strain was used. The resistance results were interpreted using the clinical breakpoints of the guidelines.

### Statistical analysis

Data entry and cleaning was done using Microsoft Excel Version 2108. Univariate analysis yielded descriptive results presented as charts for prevalence and resistance profiles using Microsoft excel. Thereafter, the data was run for bivariate analysis using Chi-square of association, followed by multivariate logistic regression using Stata 15 software. The questionnaire data was linked to laboratory results for AMR bacteria such as *E. coli*,* K. pneumoniae* and *Enterococcus* species using farm IDs. Potential associations were analyzed using logistic regression models, adjusting for confounders such as farm size and location.

## Results

### Prevalence of isolated bacteria

The most prevalent bacteria isolated was *E.* coli (98.9%), *Enterococcus* spp. (39.6%) and *K. pneumoniae* (11%). The prevalence of the bacterial isolates based on sample types and location where the samples were sourced is highlighted in Table 2.

**Table 2:** Prevalence of bacterial isolates based on sample type and location

Location	Bacteria spp.	Sample type	*n*	Bacterial prevalence (%)	95% CI
Kameza	*E. coli*	Chicken	8	8 (100%)	100% - 100%
		Human	2	2 (100%)	100% - 100%
		Environment	6	5 (83.33%)	83.03% - 83.63%
	*K. pneumoniae*	Chicken	8	2 (25%)	24.7% - 25.3%
		Human	2	0 (0%)	0% - 0%
		Environment	6	3 (50%)	49.6% - 50.4%
	*Enterococcus* spp	Chicken	8	8 (100%)	100% - 100%
		Human	2	1 (50%)	49.31% - 50.69%
		Environment	6	5 (83.33%)	83.03% - 83.63%
Chirimba	*E. coli*	Chicken	3	2 (66.67%)	66.14% - 67.2%
		Human	1	1 (100%)	100% - 100%
		Environment	2	2 (100%)	100% - 100%
	*K. pneumoniae*	Chicken	3	1 (33.33%)	32.8% - 33.86%
		Human	1	1 (100%)	100% - 100%
		Environment	2	2 (100%)	100% - 100%
	*Enterococcus* spp	Chicken	3	2 (66.67%)	66.14% - 67.2%
		Human	1	0 (0%)	0% - 0%
		Environment	2	2 (100%)	100% - 100%
Machinjiri	*E. coli*	Chicken	14	13 (92.86%)	92.73% - 92.99%
		Human	4	3 (75%)	74.58% - 75.42%
		Environment	9	8 (88.89%)	88.68% - 89.1%
	*K. pneumoniae*	Chicken	14	3 (21.43%)	21.22% - 21.64%
		Human	4	1 (25%)	24.58% - 52.42%
		Environment	9	7 (77.78%)	77.51% - 78.05%
	*Enterococcus* spp	Chicken	14	12 (85.71%)	85.53% - 85.89%
		Human	4	3 (75%)	74.58% - 75.42%
		Environment	9	8 (88.89%)	88.68% - 89.1%
Soche	*E. coli*	Chicken	2	2 (100%)	100% - 100%
		Human	1	1 (100%)	100% - 100%
		Environment	2	2 (100%)	100% - 100%
	*K. pneumoniae*	Chicken	2	0 (0%)	0% - 0%
		Human	1	0 (0%)	0% −0%
		Environment	2	1 (50%)	49.31% - 50.69%
	*Enterococcus* spp	Chicken	2	2 (100%)	100% - 100%
		Human	1	1 (100%)	100% - 100%
		Environment	2	2 (100%)	100% - 100%
New Naperi	*E. coli*	Chicken	5	5 (100%)	100% - 100%
		Human	2	0 (0%)	0% - 0%
		Environment	5	3 (60%)	59.57% - 60.43%
	*K. pneumoniae*	Chicken	5	0 (0%)	0% - 0%
		Human	2	0 (0%)	0% - 0%
		Environment	5	0 (0%)	0% - 0%
	*Enterococcus* spp	Chicken	5	5 (100%)	100% - 100%
		Human	2	2 (100%)	100% - 100%
		Environment	5	4 (80%)	79.65% - 80.35%
Manja	*E. coli*	Chicken	2	2 (100%)	100% - 100%
		Human	1	1 (100%)	100% −100%
		Environment	2	2 (100%)	100% - 100%
	*K. pneumoniae*	Chicken	2	0 (0%)	0% - 0%
		Human	1	0 (0%)	0% - 0%
		Environment	2	0 (0%)	0% - 0%
	*Enterococcus* spp	Chicken	2	2 (100%)	100% - 100%
		Human	1	1 (100%)	100% - 100%
		Environment	2	2 (100%)	100% −100%

*n* number samples collected

### AMR prevalence’s for *E. coli, K. pneumoniae*, and *Enterococcus* spp. across the sample types and location

The study revealed high prevalence of resistance among bacterial isolates (Table 3) with *E. coli* showing 95% − 100% resistance to ampicillin in all three sample types. Gentamicin and sulfamethoxazole-trimethoprim showed moderate to high resistance rates of (GEN: 45.45–58.82%) and (SXT: 58.82–85.71%) respectively. Notably, *Enterococcus* spp. demonstrated no resistance to ampicillin but concerning resistance to vancomycin (VAN: 81.82% in poultry, 66.67% in workers) and linezolid (LIN: 69.23% in environmental samples).

**Table 3:** AMR prevalence for each sample type and bacterial species

Bacteria spp.	Sample type	Antibiotic name	*n*	AMR prevalence (%)	95%CI
*E. coli*	Chicken	AMP	34	34 (100%)	100-100
		CEF	34	8 (23.53%)	23.39–23.55.39.55
		CIP	34	16 (47.06%)	46.89–47.09.89.09
		GEN	34	20 (58.82%)	58.66–58.85.66.85
		SXT	34	20 (58.82%)	58.66–58.85.66.85
	Farm worker	AMP	7	7 (100%)	100-100
		CEF	7	2 (28.57%)	28.24–28.70.24.70
		CIP	7	4 (57.14%)	56.78–57.28.78.28
		GEN	7	4 (57.14%)	56.78–57.28.78.28
		SXT	7	6 (85.71%)	85.46–85.81.46.81
	Environment	AMP	22	21 (95.45%)	95.37–95.47.37.47
		CEF	22	2 (9.09%)	8.97–9.12.97.12
		CIP	22	6 (27.27%)	27.09–27.31.09.31
		GEN	22	10 (45.45%)	45.25–45.50.25.50
		SXT	22	18 (81.82%)	81.66–81.85.66.85
*K. pneumoniae*	Chicken	CEF	2	0 (0%)	0-0
		CIP	2	1 (50%)	49.31–50.49.31.49
		GEN	2	1 (50%)	49.31–50.49.31.49
		SXT	2	1 (50%)	49.31–50.49.31.49
	Farm worker	CEF	2	0 (0%)	0-0
		CIP	2	1 (50%)	49.31–50.49.31.49
		GEN	2	1 (50%)	49.31–50.49.31.49
		SXT	2	1 (50%)	49.31–50.49.31.49
	Environment	CEF	14	2 (14.29%)	14.10–14.33.10.33
		CIP	14	3 (21.43%)	21.21–21.49.21.49
		GEN	14	5 (35.71%)	35.46–35.78.46.78
		SXT	14	10 (71.43%)	71.19–71.49.19.49
*Enterococcus *spp.	Chicken	AMP	22	0 (0%)	0-0
		VAN	22	18 (81.82%)	81.66–81.85.66.85
		LIN	22	11 (50%)	49.79–50.04.79.04
		CIP	22	15 (68.18%)	67.99–68.22.99.22
		TIG	22	9 (40.91%)	40.70–40.95.70.95
	Farm worker	AMP	6	0 (0%)	0-0
		VAN	6	4 (66.67%)	66.29–66.82.29.82
		LIN	6	5 (83.33%)	83.04–83.46.04.46
		CIP	6	2 (33.33%)	32.96–33.49.96.49
		TIG	6	3 (50%)	49.60–50.16.60.16
	Environment	AMP	13	0 (0%)	0-0
		VAN	13	5 (38.46%)	38.20–38.53.20.53
		LIN	13	9 (69.23%)	68.98–69.30.98.30
		CIP	13	8 (61.54%)	61.27–61.61.27.61
		TIG	13	4 (30.77%)	30.52–30.84.52.84

*AMP* ampicillin, *CEF* cefotaxime, *CIP* ciprofloxacin, *GEN* gentamicin, *SXT* trimethoprim sulfamethoxazole, *VAN* vancomycin, *LIN* linezolid, *TIG* tigecycline, *n* number of isolates

In the Table 4 (Supplementary information 2), it shows AMR prevalence of *E. coli*,* K. pneumoniae*, and *Enterococcus* species based on the location where the samples were collected. AMR prevalence in *E. coli* showed relative uniformity (80–100%) resistance in nearly all chicken and human sample locations. Variability in resistance prevalence was shown in *K. pneumoniae*, with absolute resistance (100%) to ciprofloxacin in chickens from Machinjiri and Soche and environmental isolates from Kameza, Machinjiri, Soche, and New Naperi but otherwise lower resistance to cefotaxime (0–12.5.5%) in most locations. Only in Chirimba human samples, there was 100% resistance to ciprofloxacin, gentamicin, sulfamethoxazole trimethoprim.

**Table 4:** AMR prevalence’s for each sampling location across sample types

Location	Bacteria spp.	Sample type	Antibiotic name	*n*	AMR prevalence	95% CI
Kameza	*E. coli*	Chicken	AMP	8	8(100)	100-100
			CEF	8	1(12.5)	12.27–12.73.27.73
			CIP	8	4(50)	49.65–50.35.65.35
			GEN	8	5(62.5)	62.16–62.84.16.84
			SXT	8	8(100)	100-100
		Human	AMP	2	2(100)	100-100
			CEF	2	0	0-0
			CIP	2	1(50)	49.31–50.69.31.69
			GEN	2	1(50)	49.31–50.69.31.69
			SXT	2	2(100)	100-100
		Environment	AMP	5	5(100)	100-100
			CEF	5	1(20)	19.65–20.35.65.35.65.35
			CIP	5	5(100)	100-100
			GEN	5	2(40)	39.57–40.43.57.43
			SXT	5	4(80)	79.65–80.35.65.35
	*K. pneumoniae*	Chicken	CEF	1	0	0-0
			CIP	1	0	0-0
			GEN	1	0	0-0
			SXT	1	0	0-0
		Human	CEF	1	0	0-0
			CIP	1	0	0-0
			GEN	1	0	0-0
			SXT	1	0	0-0
		Environment	CEF	2	0	0-0
			CIP	2	2(100)	100-100
			GEN	2	0	0-0
			SXT	2	1(50)	49.31–50.69.31.69
Machinjiri	*E. coli*	Chicken	AMP	14	14(100)	100-100
			CEF	14	3(21.43)	21.21–21.64.21.64
			CIP	14	4(28.57)	28.33–28.81.33.81
			GEN	14	7(50)	49.74–50.26.74.26
			SXT	14	14(100)	100-100
		Human	AMP	3	3(1000	100-100
			CEF	3	1(33.33)	32.8–33.87.8.87
			CIP	3	1(33.33)	32.8–33.87.8.87
			GEN	3	2(66.67)	66.13–67.2.13.2
			SXT	3	3(100)	100-100
		Environment	AMP	7	6(85.71)	85.46–85.97.46.97
			CEF	7	0	0-0
			CIP	7	2(28.57)	28.24–28.91.24.91
			GEN	7	3(42.86)	42.49–43.22.49.22
			SXT	7	7(100)	100-100
	*K. pneumoniae*	Chicken	CEF	1	0	0-0
			CIP	1	1(100)	100-100
			GEN	1	1(100)	100-100
			SXT	1	1(100)	100-100
		Human	CEF	1	0	0-0
			CIP	1	0	0-0
			GEN	1	0	0-0
			SXT	1	0	0-0
		Environment	CEF	8	1(12.5)	12.27–12.73.27.73
			CIP	8	3(37.5)	37.16–37.84.16.84
			GEN	8	2(25)	24.7–25.3.7.3
			SXT	8	6(75)	74.7–75.3.7.3
	*Enterococcus *spp.	Chicken	AMP	13	0	0-0
			VAN	13	12(92.31)	92.16–92.45.16.45
			LIN	13	7(53.85)	53.58–54.12.58.12
			CIP	13	8(61.54)	61.27–61.8.27.8
			TIG	13	5(38.46)	38.2–38.73.2.73
		Human	AMP	3	0	0-0
			VAN	3	3(100)	100-100
			LIN	3	3(100)	100-100
			CIP	3	1(33.33)	32.8–33.87.8.87
			TIG	3	2(66.67)	66.13–67.2.13.2
		Environment	AMP	5	0	0-0
			VAN	5	2(40)	39.57–40.43.57.43
			LIN	5	3(60)	59.57–60.43.57.43
			CIP	5	3(60)	59.5760.43
			TIG	5	2(40)	39.57–40.43.57.43
Chirimba	*E. coli*	Chicken	AMP	3	3(100)	100-100
			CEF	3	2(66.67)	66.13–67.2.13.2
			CIP	3	2(66.67)	66.1367.2
			GEN	3	3(100)	100-100
			SXT	3	3(100)	100-100
		Human	AMP	1	1(100)	100-100
			CEF	1	0	0-0
			CIP	1	1(100)	100-100
			GEN	1	1(100)	100-100
			SXT	1	1(100)	100-100
		Environment	AMP	2	2(100)	100-100
			CEF	2	0	0-0
			CIP	2	0	0-0
			GEN	2	1(50)	49.31–50.69.31.69
			SXT	2	2(100)	100-100
	*K. pneumoniae*	Chicken	CEF	1	0	0-0
			CIP	1	0	0-0
			GEN	1	0	0-0
			SXT	1	0	0-0
		Human	CEF	1	0	0-0
			CIP	1	1(100)	100-100
			GEN	1	1(100)	100-100
			SXT	1	1(100)	100-100
		Environment	CEF	2	0	0-0
			CIP	2	0	0-0
			GEN	2	1(50)	49.31–50.69.31.69
			SXT	2	1(50)	49.31–50.69.31.69
	*Enterococcus *spp.	Chicken	AMP	2	0	0-0
			VAN	2	2(100)	100-100
			LIN	2	1(50)	49.31–50.69.31.69
			CIP	2	1(50)	49.31–50.69.31.69.31.69
			TIG	2	1(50)	49.31–50.69.31.69
		Human	AMP	1	0	0-0
			VAN	1	0	0-0
			LIN	1	1(100)	100-100
			CIP	1	1(100)	100-100
			TIG	1	0	0-0
		Environment	AMP	2	0	0-0
			VAN	2	2(100)	100-100
			LIN	2	2(100)	100-100
			CIP	2	1(50)	49.31–50.69.31.69
			TIG	2	0	0-0
Soche	*E. coli*	Chicken	AMP	2	2(100)	100-100
			CEF	2	0	0-0
			CIP	2	1(50)	49.31–50.69.31.69
			GEN	2	2(100)	100-100
			SXT	2	2(100)	100-100
		Human	AMP	1	0	0-0
			CEF	1	0	0-0
			CIP	1	0	0-0
			GEN	1	0	0-0
			SXT	1	0	0-0
		Environment	AMP	2	2(100)	100-100
			CEF	2	0	0-0
			CIP	2	0	0-0
			GEN	2	2(100)	100-100
			SXT	2	2(100)	100-100
	*K. pneumoniae*	Chicken	CEF	1	0	0-0
			CIP	1	0	0-0
			GEN	1	0	0-0
			SXT	1	1(100)	100-100
		Human	CEF	1	0	0-0
			CIP	1	0	0-0
			GEN	1	0	0-0
			SXT	1	0	0-0
		Environment	CEF	2	0	0-0
			CIP	2	0	0-0
			GEN	2	1(50)	49.31–50.69.31.69
			SXT	2	1(50)	49.31–50.69.31.69
	*Enterococcus *spp.	Chicken	AMP	2	0	0-0
			VAN	2	2(100)	100-100
			LIN	2	1(50)	49.31–50.69.31.69
			CIP	2	2(100)	100-100
			TIG	2	1(50)	49.31–50.69.31.69
		Human	AMP	1	0	0-0
			VAN	1	0	0-0
			LIN	1	0	0-0
			CIP	1	0	0-0
			TIG	1	0	0-0
		Environment	AMP	1	0	0-0
			VAN	1	1(100)	100-100
			LIN	1	1(100)	100-100
			CIP	1	1(100)	100-100
			TIG	1	0	0-0
New Naperi	*E. coli*	Chicken	AMP	5	5(100)	100-100
			CEF	5	2(40)	39.57–40.43.57.43
			CIP	5	3(60)	59.57–60.43.57.43
			GEN	5	2(40)	39.57–40.43.57.43
			SXT	5	5(100)	100-100
		Human	AMP	1	0	0-0
			CEF	1	0	0-0
			CIP	1	0	0-0
			GEN	1	0	0-0
			SXT	1	0	0-0
		Environment	AMP	4	4(100)	100-100
			CEF	4	1(25)	24.58–25.42.58.42
			CIP	4	1(25)	24.58–25.42.58.42
			GEN	4	2(50)	49.51–50.49.51.49
			SXT	4	3(75)	74.58-74.42.58.42.58.42.58.42
	*K. pneumoniae*	Chicken	CEF	1	0	0-0
			CIP	1	0	0-0
			GEN	1	0	0-0
			SXT	1	0	0-0
		Human	CEF	1	0	0-0
			CIP	1	0	0-0
			GEN	1	0	0-0
			SXT	1	0	0-0
		Environment	CEF	1	1(100)	100-100
			CIP	1	0	0-0
			GEN	1	1(100)	100-100
			SXT	1	1(100)	100-100
	*Enterococcus *spp.	Chicken	AMP	3	0	0-0
			VAN	3	1(33.33)	32.8–33.87.8.87
			LIN	3	1(33.33)	32.8–33.87.8.87
			CIP	3	1(100)	100-100
			TIG	3	1(33.33)	32.8–33.87.8.87
		Environment	AMP	1	0	0-0
			VAN	1	1(100)	100-100
			LIN	1	1(100)	100-100
			CIP	1	0	0-0
			TIG	1	0	0-0
		Human	AMP	3	0	0-0
			VAN	3	0	0-0
			LIN	3	3(100)	100-100
			CIP	3	3(100)	100-100
			TIG	1	1(100)	100-100
Manja	*E. coli*	Chicken	AMP	2	2(100)	100-100
			CEF	2	0	0-0
			CIP	2	2(100)	100-100
			GEN	2	1(50)	49.31–50.69.31.69
			SXT	2	1(50)	49.31–50.69.31.69
		Environment	AMP	1	1(100)	100-100
			CEF	1	1(100)	100-100
			CIP	1	1(100)	100-100
			GEN	1	0	0-0
			SXT	1	0	0-0
		Human	AMP	2	2(100)	100-100
			CEF	2	0	0-0
			CIP	2	2(100)	100-100
			GEN	2	0	0-0
			SXT	2	0	0-0
	*K. pneumoniae*	Chicken	CEF	1	0	0-0
			CIP	1	0	0-0
			GEN	1	0	0-0
			SXT	1	0	0-0
		Environment	CEF	1	0	0-0
			CIP	1	0	0-0
			GEN	1	0	0-0
			SXT	1	0	0-0
		Human	CEF	1	0	0-0
			CIP	1	0	0-0
			GEN	1	0	0-0
			SXT	1	0	0-0
	*Enterococcus*spp.	Chicken	AMP	2	0	0-0
			VAN	2	1(50)	49.31–50.69.31.69.31.69
			LIN	2	1(50)	49.31
			CIP	2	1(50)	49.31–50.69.31.69.31.69
			TIG	2	1(50)	49.31–50.69.31.69.31.69
		Environment	AMP	1	0	0-0
			VAN	1	0	0-0
			LIN	1	0	0-0
			CIP	1	0	0-0
			TIG	1	1(100)	100-100
		Human	AMP	2	0	0-0
			VAN	2	0	0-0
			LIN	2	0	0-0
			CIP	2	0	0-0
			TIG	2	1(50)	49.31–50.69.31.69

*AMP* ampicillin, *CEF* cefotaxime, *CIP* ciprofloxacin, *GEN* gentamicin, *SXT* trimethoprim sulfamethoxazole, *VAN* vancomycin, *LIN* linezolid, *TIG* tigecycline, *n* number of isolates

### Resistance profiles in *E. coli*,* K. pneumoniae* and *Enterococcus* species

#### Resistance profiles in *E. coli*

**Fig. 2:**
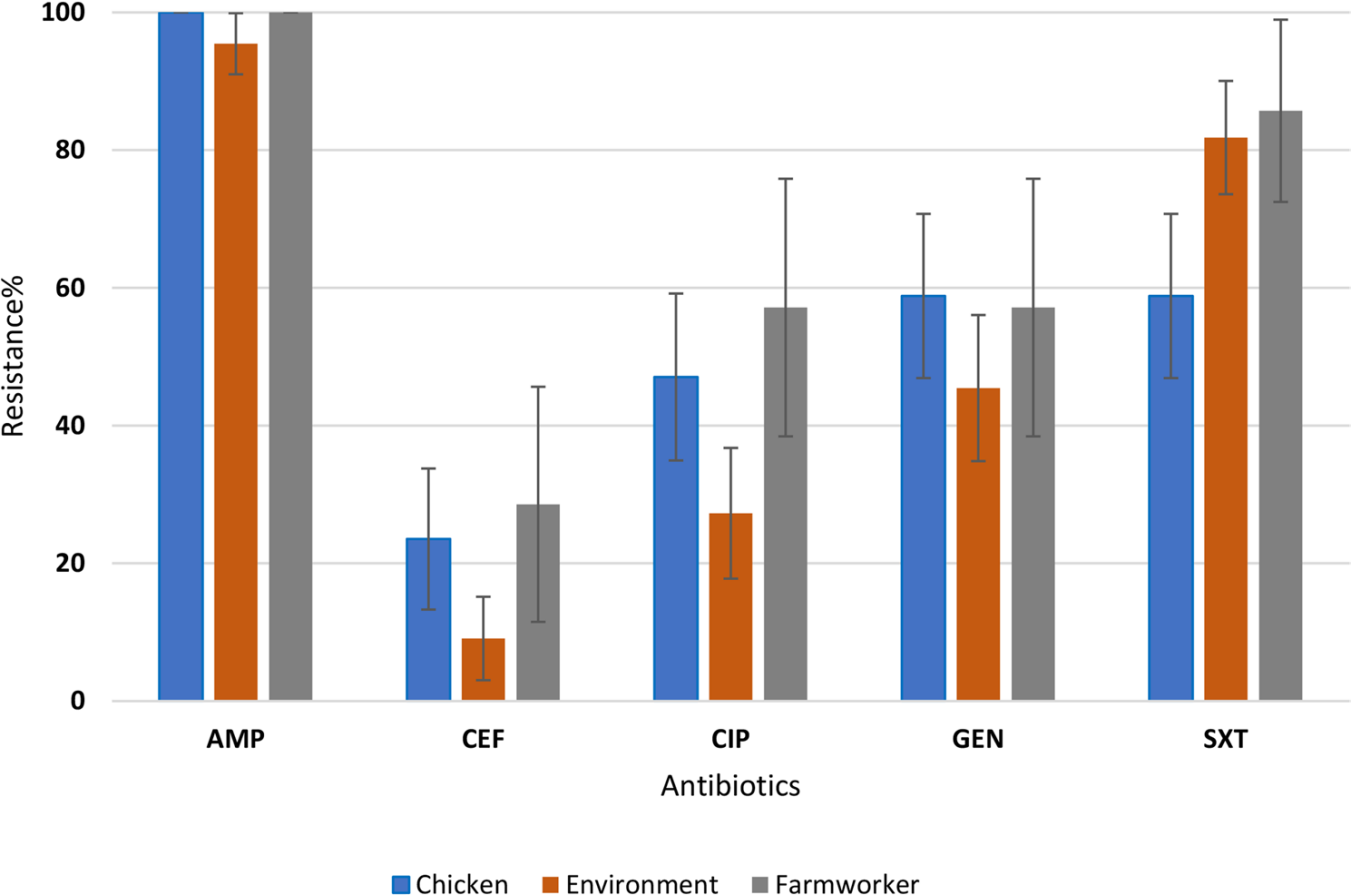
*E. coli* resistance in chickens, farm workers and farm dust. AMP: Ampicillin, CXT: Cefotaxime, CIP: Ciprofloxacin, GEN: Gentamicin, SXT: Sulfamethoxazole Trimethoprim

The resistance profiles of *E. coli* for the three sample types in Fig. 2 show no significant difference in resistance profiles across the three sample types to ampicillin (*p* = 0.128), cefotaxime (*p* = 0.322), ciprofloxacin (*p* = 0.166), gentamicin (*p* = 0.53), and sulfamethoxazole trimethoprim (*p* = 0.079).

#### Resistance profiles for *K. pneumoniae*

**Fig. 3:**
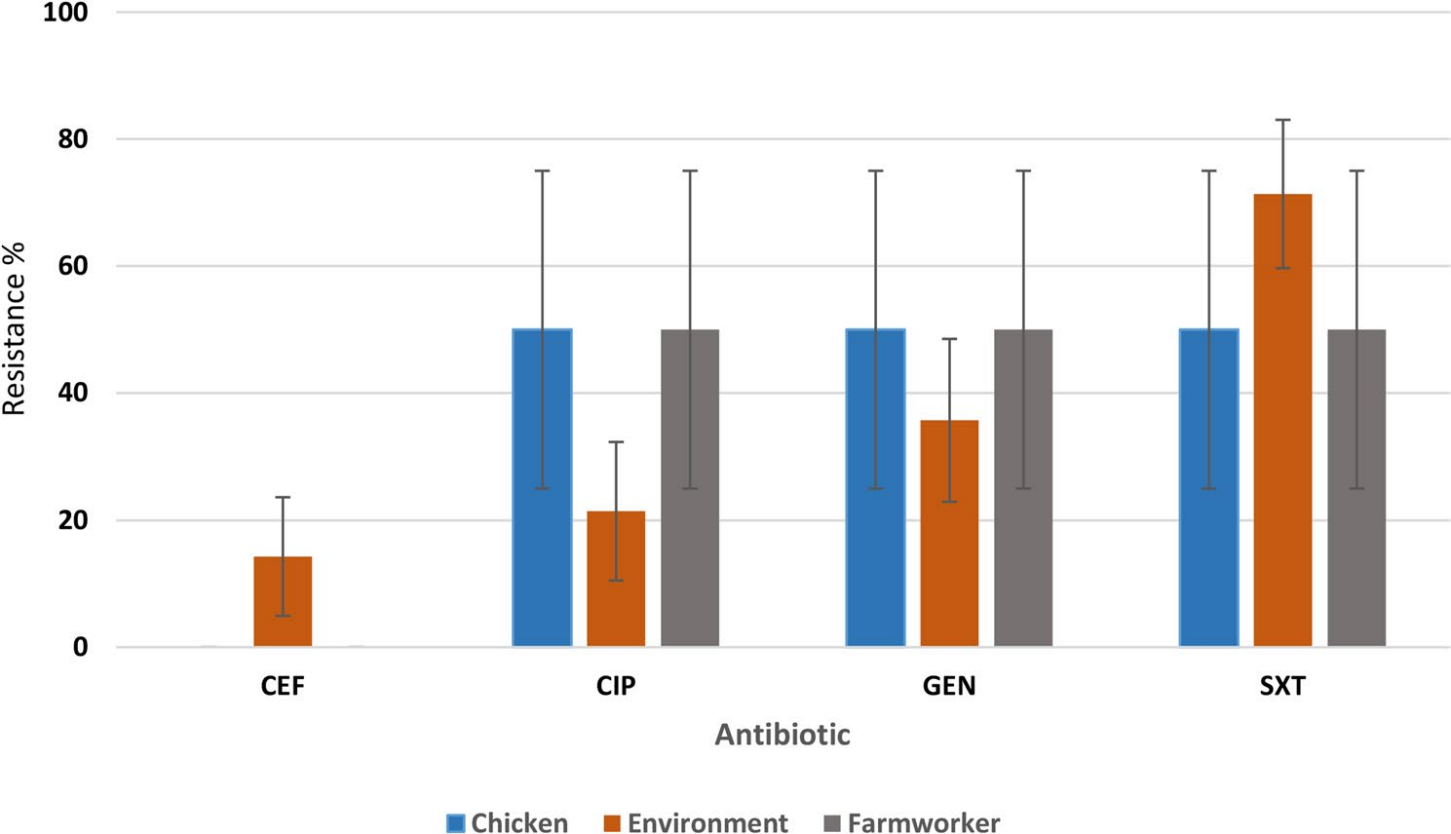
*K. pneumoniae* resistance in chickens, farm workers and farm dust. CXT: Cefotaxime, CIP: Ciprofloxacin, GEN: Gentamicin, SXT: Sulfamethoxazole Trimethoprim

The resistance profiles for *K. pneumoniae* were 71% in environmental samples for sulfamethoxazole trimethoprim unlike resistance profiles for human and chicken samples (Fig. 3). The sample size was however too small for statistical comparison.

#### Resistance profiles for *Enterococcus* species

**Fig. 4:**
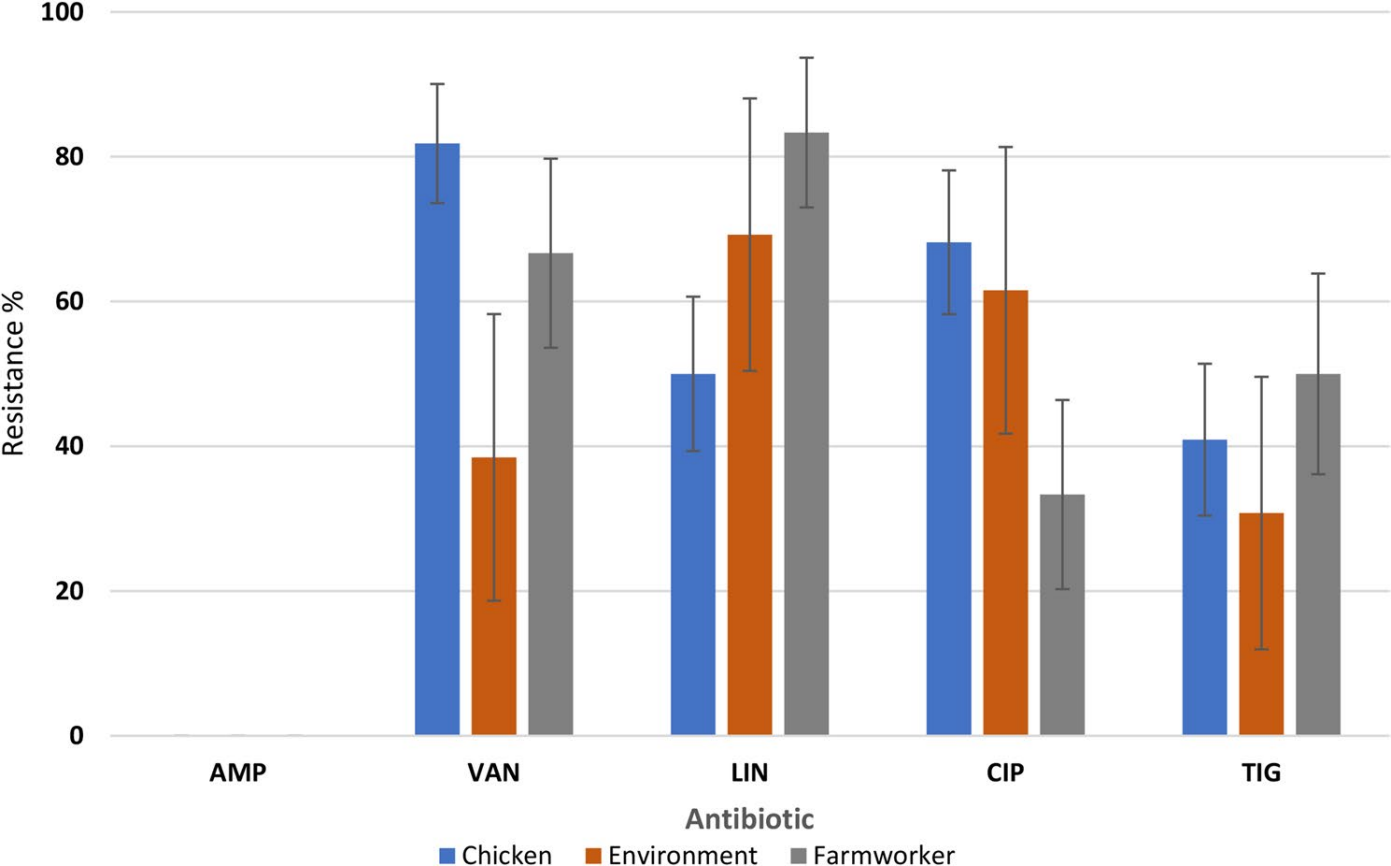
*Enterococcus* species resistance in chickens, farm workers and farm dust. AMP: Ampicillin, VAN: Vancomycin, LIN: Linezolid, CIP: Ciprofloxacin, TIG: Tigecycline

The resistance profiles of *Enterococcus* species in Fig. 4 showed no significant differences in resistance among the sample types to linezolid (*p* = 0.214), ciprofloxacin (*p* = 0.141), and tigecycline (*p* = 0.508). There is a statistical difference across the sample types in resistance to vancomycin (*p* = 0.028).

### Comparison of resistance patterns between broiler and layer farms

The study found no significant differences in resistance patterns of *E. coli* to ampicillin, and sulfamethoxazole trimethoprim between samples from broiler farms and layer farms (*p* = 0.76).

The Fig. 6 compares the resistance patterns of *K. pneumoniae* in broiler farms and layer farms. There were no significant differences in resistance patterns between samples from broiler farms and layer farms (*p* = 0.99).

Even though the resistance patterns of *Enterococcus* species are varied across sample types in Fig. 7. there were no significant differences in resistance patterns between samples from broiler farms and layer farms (*p* = 0.83).

### Potential risk factors associated with the presence of resistant bacteria in layer and broiler farms in Blantyre city

After running linear regression test for the 9 variables against AMR status, as shown in Tables 3 and 5, farm category, withdrawal periods and waste managements were the variables with *p*-value < 0.05.

**Table 5:** Unadjusted potential risk factor

AMR status	Odds Ratio	Std. Err.	Z	*P*>z	[95% Conf.	Interval]
Farm type	0.738	0.466	−0.48	0.630	0.214	2.544
Farm category	0.208	0.192	−1.92	0.028	0.042	1.034
Contact frequency	1.160	0.624	0.28	0.783	0.404	3.327
Withdrawal periods	5.317	2.841	3.07	0.013	1.892	14.941**
AMR knowledge	0.923	0.932	−0.08	0.937	0.127	6.683
Antimicrobial disposal	0.628	0.218	−1.34	0.180	0.317	1.241
PPE used	0.730	0.546	−0.42	0.674	0.169	3.163
Drug sachets disposal	1.543	0.596	1.12	0.262	0.724	3.290
Waste management	3.415	1.826	2.33	0.037	1.216	9.589**
_cons	1.148	0.781	0.21	0.430	0.311	4.236

** significant risk factors

## Discussion

The current study has revealed high resistance profiles and associated risk factors of *E. coli*,* K. pneumoniae*, and *Enterococcus* species from the human, poultry and environment samples. These indicate increasing trends of AMR burdens at human livestock and environment interface. This is a concern to the public health and livestock production which requires immediate intervention. Further, the study has revealed that no withdraw period and poor disposal of wastes are drivers of AMR emergence and spread among the small and medium scale poultry farmers. These results call for collective action using a One Health approach to mitigate the emergence of AMR at source [18].

There is no significant difference in *E. coli* resistance among farm workers, poultry house dust, and poultry as shown in Fig. 2. This is in agreement with findings from other studies from Zambia and Tanzania [25, 26]. This could be because the small and medium scale farmers are practicing similar type of management which does not observe withdraw periods and there is poor disposal of farm wastes as previously reported by Da Costa [8]. In Tanzania, *E. coli* isolates from poultry farms were highly resistant to sulfamethoxazole trimethoprim which is also commonly used antibiotic in poultry [26]. The farm workers who handled poultry also harbored resistant *E. coli*, focusing on occupational exposure and the intimate relationship between humans, animals, and the environment [6]. In *K. pneumoniae* (Fig. 3), the high resistance found in poultry compared to poultry house dust and farm workers could be a sign of increased antibiotic pressure in poultry production. For instance, antibiotics are used in feed or as growth promoters. Further, this could suggest the possible spread of the resistant bacteria from source where antibiotics are applied to the surrounding. A significant resistance in *K. pneumoniae* among the poultry isolates was revealed in an Ethiopian study, where some of the isolates were multidrug-resistant [27]. Globally, *K. pneumoniae* has been documented as a priority AMR in poultry production systems. In European reports, for instance, there is high resistance among poultry *K. pneumoniae*, particularly to beta-lactams and quinolones, and also to moderate resistance among environmental dust and human samples [28].

Blantyre’s high level of AMR among the poultry farms is at 87.3% for *E. coli* and 29.6% for *K. pneumoniae* closely follows Nigeria and Kenya findings [29]. These findings showed *E. coli* (82%) and *K. pneumoniae* (34%) with higher in poultry samples under the same driver of antibiotic misuse. Levels of resistance from these studies concur with findings of this study which suggest poultry to be the key reservoir of resistant bacteria. It may also suggest that poultry farms are likely sites for the emergency of resistant bacteria species.

The trends of resistance for *Enterococcus species* to different antibiotics and sample sources (chicken, environment, and human) in Fig. 4, are alarming considering the transmission of AMR at the interface of human, animal, and environment. *Enterococcus species* patterns of resistance illustrated in this study are similar to a study that was carried out in Zambia which exhibited some level of resistance against antibiotics that were subjected to testing [30]. Comparatively, the current study reports no resistance profile for ampicillin at 100% susceptibility than the study conducted in Zambia which was 68% [30]. This difference could be due to different levels of hygiene which prevents over-dependence of antibiotics to prevent diseases as previously reported by Founou et al. [31]

The high levels of vancomycin resistance in *Enterococcus* isolates, especially in human (80%) and chicken samples (81.8%), align with global concerns of vancomycin-resistant Enterococcus (VRE). For instance, human health VRE is a leading cause of hospital-acquired infections and is linked to high morbidity and mortality rates. A study in South Africa reported vancomycin resistance in Enterococcus isolates from poultry and farm workers, highlighting occupational exposure as a transmission route [32]. Similarly, in Kenya, vancomycin resistance was prevalent in isolates from both humans and poultry, reinforcing the role of poultry farms as reservoirs of VRE [33, 34]. Linezolid resistance also observed primarily in human isolates raises (100%) alarm as this antibiotic is a last-resort drug for treating VRE infections. Resistance in poultry and environmental samples suggests that this resistance trait might spread from human use to other reservoirs [35].

**Fig. 5:**
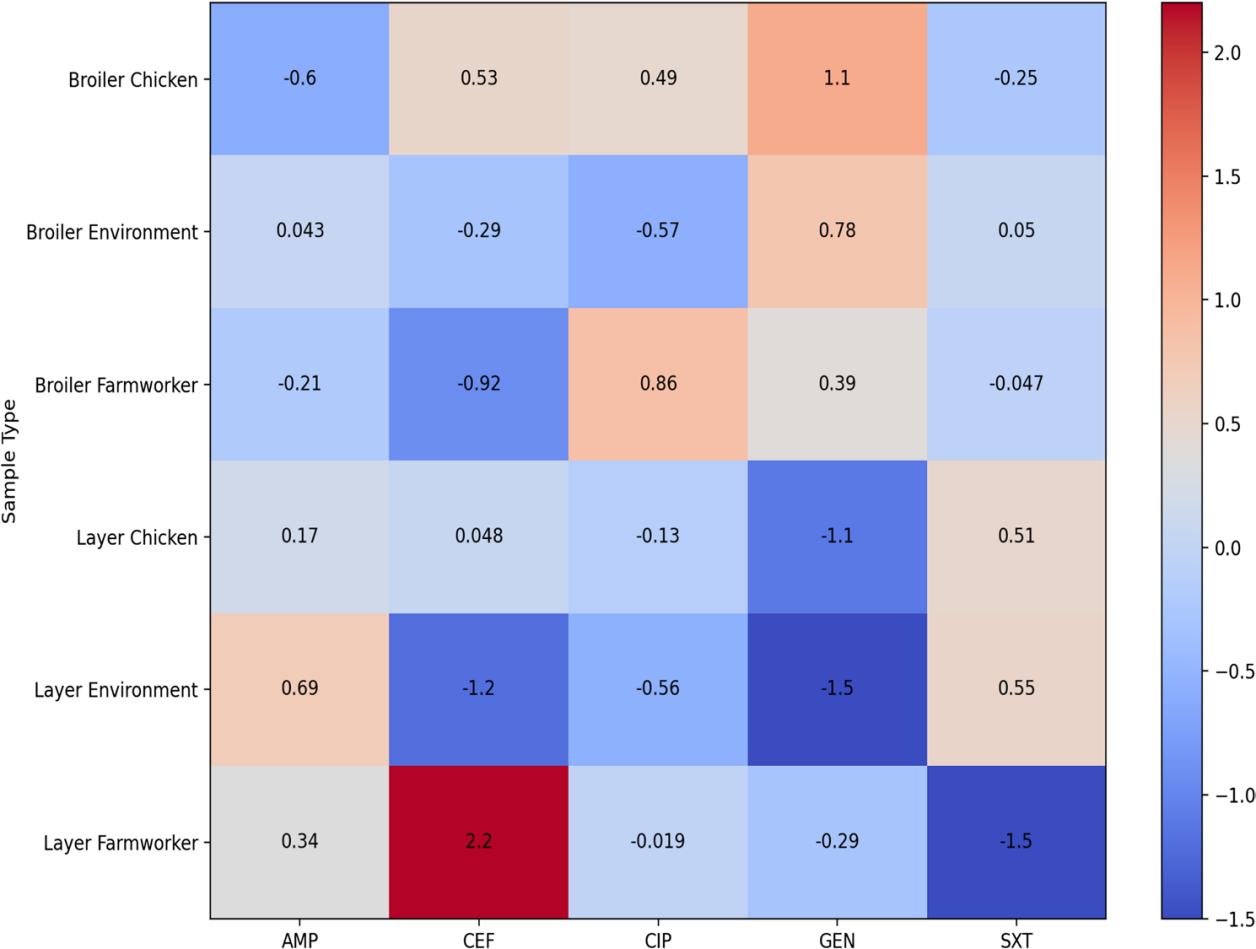
Resistance patterns of *E. coli* in broiler and layer farms. AMP: Ampicillin, CEF: Cefotaxime, CIP: Ciprofloxacin, GT: Gentamycin, SXT: Sulfamethoxazole Trimethoprim

A Tanzanian study reported similar patterns of resistance of *E. coli* (Fig. 5), with highest rates of sulfonamides in poultry and farm environments [26]. Another study in Nigeria reported the poultry litter as a reservoir of highly resistant *E. coli*, which could readily be transferred to humans and other environments surrounding [36]. There is resistance pattern variation in cefotaxime, ciprofloxacin, and gentamicin that exists more on broiler than layer farms. This is in agreement with another research study [37] that reported high levels of resistance on broiler compared to layer farms in Nepal. This is could be due to extensive use of antibiotics for growth promotion and disease prevention within a short period. Avian pathogenic *E. coli* showed 99.4% resistance to ampicillin and 92% resistance to enrofloxacin which is a concern in livestock production. This could also pose a threat to humans in close proximity with the chickens.

**Fig. 6:**
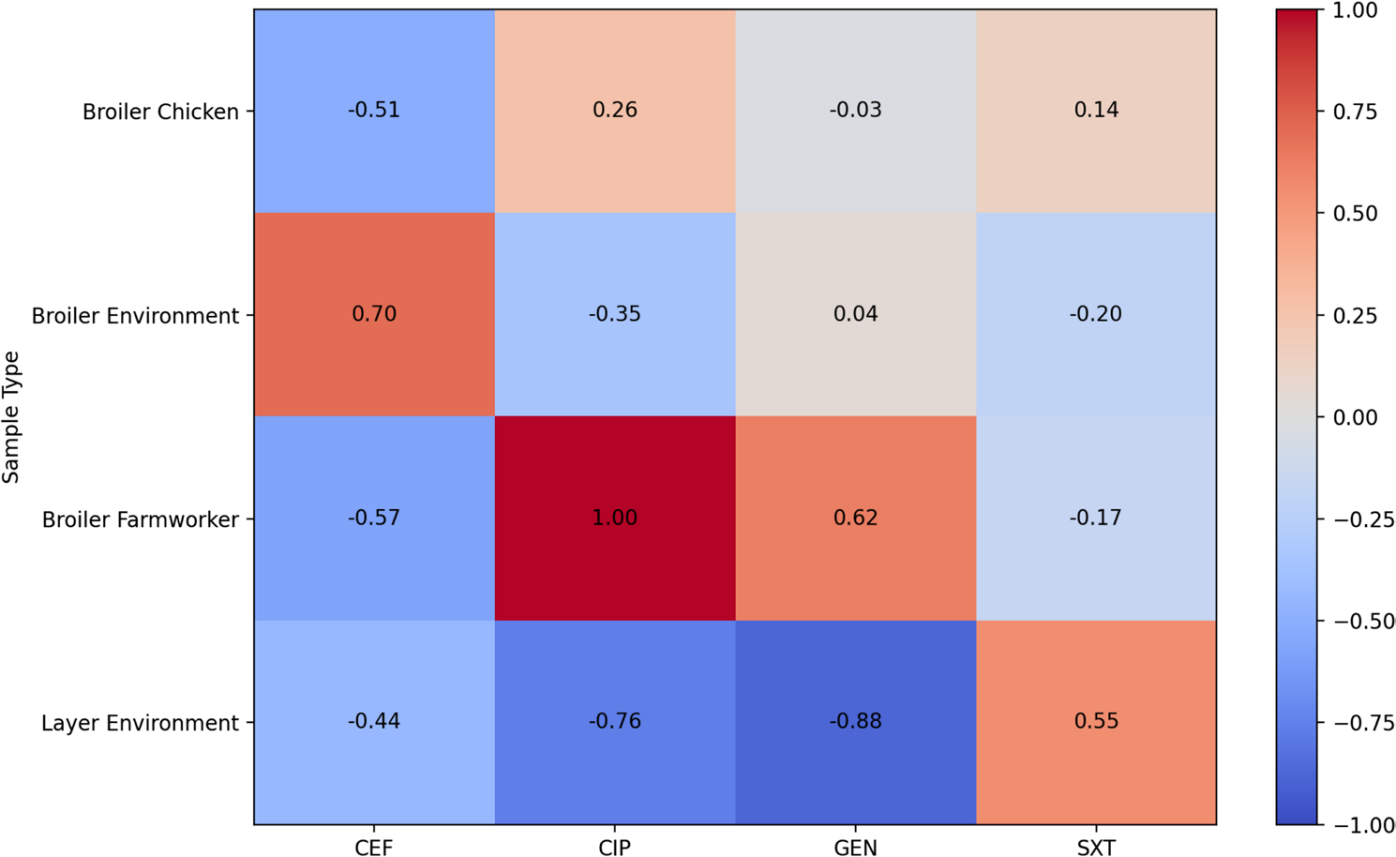
Resistance patterns of *K. pneumoniae* in broiler and layer farm. CEF: Cefotaxime, CIP: Ciprofloxacin, GT: Gentamycin, SXT: Sulfamethoxazole Trimethoprim. Broiler Human: House attendant of broiler farm house, Layer Human: House attendant of layer farm house

Figure 6 shows that *K. pneumoniae* resistance patterns vary across sample types. Strong positive associations were observed for ciprofloxacin (1.0) in broiler human samples and cefotaxime (0.7) in broiler environmental samples while strong negative associations were observed for ciprofloxacin (−0.76) and gentamicin (−0.088) in layer environmental samples. In comparison, broiler production system is characterized by production cycles, higher stocking densities and intensive antibiotic use within a short period of time compared to layer production system. For instance, *K. pneumoniae* isolates from broilers showed high levels of multidrug resistance, with resistance levels over 82% for ciprofloxacin and considerable resistance to other widely used antibiotics in another study [38]. High AMR prevalence in poultry farms poses zoonotic risks, especially for farm workers that had resistant *E. coli* and *Enterococcus species* captured by hand swabs. Resistant bacteria also found in dust suggest environmental dissemination, potentially contaminating water and soil thereby affecting nearby communities.

In addition, layer chickens tend to live longer and are less exposed to antibiotics, hence the potential for reduced patterns and profiles as of resistance as compared to broiler chickens. This difference in exposure to antibiotics accounts for the observed difference in the resistance profiles of broiler and layer farms in tandem with trends reported in Tanzania [39], where farms for layers had lower rates of resistance due to reduced antimicrobial pressure. The detection of resistance in environmental samples from both broiler and layer farms suggests a higher risk of cross-contamination and environmental transmission in poultry systems. This concurs with Singh & Kim [40], who reported that environmental samples in the vicinity of poultry farms have higher resistance due to runoff and irresponsible waste disposal. This suggested the interconnectedness that exist between poultry and environment and the potential of spillover of resistance bacteria from poultry to the environment.

**Fig. 7:**
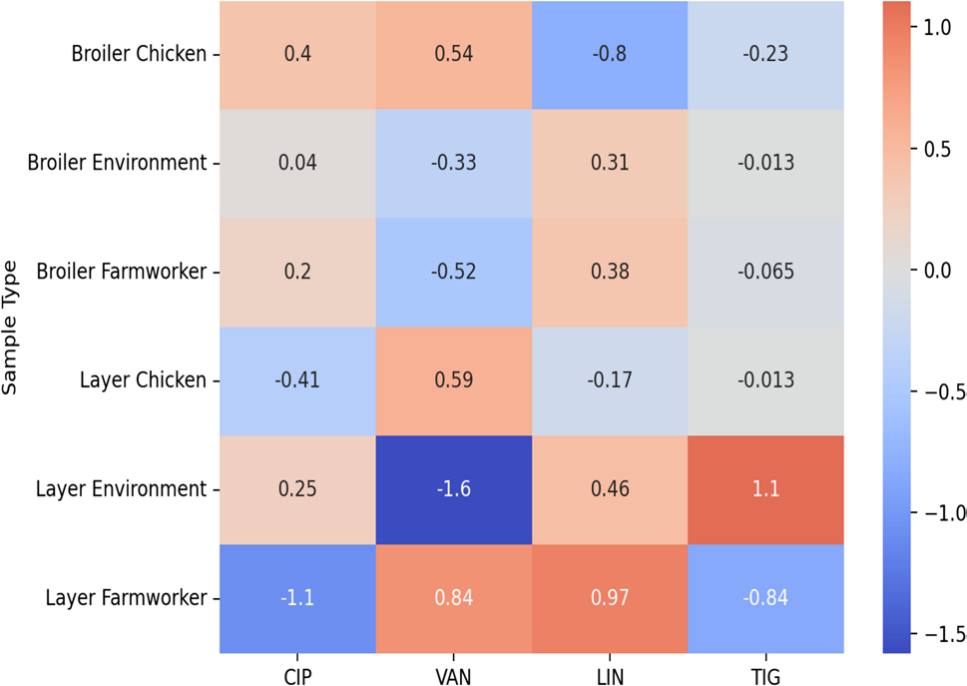
Resistance patterns of *Enterococcus species* in broiler and layer farm. CIP: Ciprofloxacin, LIN: Linezolid, TIG: Tigecycline, VAN: Vancomycin

This study reports high levels of resistance to vancomycin in chicken and human isolates (Fig. 7), while the environmental isolates have comparatively low rates. This presents the potential threat to public health due to the significance of vancomycin-resistant Enterococcus (VRE). It is known that VRE is one of the leading etiologies of nosocomial infections in humans and poultry could be considered as potential source of the challenge. Resistance to linezolid is more frequently observed in human isolates, suggesting selective pressure from clinical use, but its presence in chicken and environmental isolates is a public health concern as it suggests that poultry are becoming sites for the emergence of resistant bacterial species to the critical antibiotics in human medicine. The reduced resistance of tigecycline across all sources aligns with global patterns where tigecycline remains effective against multidrug-resistant Enterococcus. A South African study reported extensive VRE prevalence in poultry and human farm workers, with occupational exposure being an important transmission mode [31], which could equally apply in this study. Kenyan studies corroborated vancomycin and linezolid resistance profiles, with farms acting as reservoirs for resistant poultry strains [34].

The identified significant risk factors associated with AMR status among poultry farms in Blantyre city have been highlighted in Table 5. Notably, the absence of proper withdrawal periods (OR = 5.32, 95% CI: 1.89–14.94, *p* = 0.013) and inadequate waste management practices (OR = 3.42, 95% CI: 1.22–9.59, *p* = 0.037) were strongly associated with increased odds of AMR detection. This could be due to limited interactions between animal health officers as the drug sellers rarely consult experts on AMU [11, 20]. These findings are in line with findings from a review conducted in China that poor waste management contribute to the persistence and dissemination of resistant bacteria in poultry environments [39]. Another study conducted in Quebec in Canada have shown the negative impacts of short-term antibiotic withdrawal and antibiotic overuse on AMR occurrence resulting into lower production performance [41]. Non-compliance to proper withdrawal periods, may led to antibiotic residues persisting in poultry meat, eggs, and the environment, leading to their presence in foodstuffs and increased AMR risks. Continued presence of such residues in the food chain has direct implications for public health through the facilitation of transmission of resistant bacteria to humans through ingestion or environmental contact.

## Conclusion

The findings of this study highlight the alarming prevalence of AMR among *E. coli*,* K. pneumoniae*, and *Enterococcus species* in broiler and layer farms in Blantyre, Malawi. The reported AMR profiles are of public health concern and require awareness campaigns among poultry farmers to manage the threat. The uniform resistance profiles across human, poultry and environmental samples suggest a dynamic transmission of resistant bacteria at the human-animal-environment interface, thus emphasizing the need for a One health approach to mitigate AMR.

## Limitations of the study

Even though this study provides critical insights on AMR in poultry farms in Blantyre, the following limitations should be considered when interpreting the findings. The applicability of the breakpoints for interpreting *Enterococcus* species resistance is less certain as highlighted in the EUCAST guideline that was used for interpretation. Due to budgetary limitations, only 11 of 123 farms were sampled for laboratory analysis, hence potentially limiting effect on the generalizability of findings of this study. The study was confined to the Blantyre urban and peri-urban area hence the findings may not be representative of AMR trends in rural or other urban Malawi locations. As a cross-sectional study, it captures prevalence at a single time point, thereby preventing assessment of temporal trends. Data on antibiotic usage and farm practices relied on farmer recall, which may be subject to social desirability bias or underreporting of non-compliant practices such as antibiotic misuse.

## Supplementary Information

Supplementary Material 1.

Supplementary Material 2.

Supplementary Material 3.

Supplementary Material 4.

## Data Availability

The data supporting the findings is available upon request, subject to maintaining participant confidentiality.
