# Supplementary material for: Antimicrobial susceptibility of Escherichia coli, Klebsiella pneumoniae, and Enterococcus species and the associated risk factors in poultry farms in Blantyre City: a wake-up call to the one health approach
Source: BMC Vet Res. 2025 Dec 23;22:73. doi: 10.1186/s12917-025-05189-7 (PMC12882281; doi:10.1186/s12917-025-05189-7)
Supplement: Supplementary file 1 — Supplementary Material 1. [file 12917_2025_5189_MOESM1_ESM.docx]

**
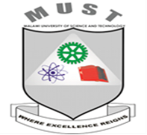
**

**Participant Information Leaflet and Consent Form**

| **Title of Research Project:** | |
| --- | --- |
| **PREVALENCE AND ANTIMICROBIAL RESISTANCE PATTERNS OF *ESCHERICHIA COLI* AND *KLEBSIELLA PNEUMONIAE* IN POULTRY FARMS IN BLANTYRE** | |
| **DETAILS OF PRINCIPAL INVESTIGATOR (PI):** | |
| **Title, first name, surname: MISS MUONAOUZA DELEZA** | **MUSTREC reference number: P.04/2024/127** |
| **Full postal and email address: P. O. BOX 1525, BLANTYRE**  **m**[**oh-010-22@must.ac.mw**](mailto:oh-010-22@must.ac.mw) | **PI Contact number:**  **0884654335** |

We would like to invite you to take part in a research project. Please take some time to read the information presented here, which will explain the details of this project. Please ask the study staff or doctor any questions about any part of this project that you do not fully understand. It is very important that you are completely satisfied that you clearly understand what this research entails and how you could be involved. Also, your participation is **entirely voluntary,** and you are free to decline to participate. In other words, you may choose to take part, or you may choose not to take part. Nothing bad will come of it if you say no: it will not affect you negatively in any way whatsoever. Refusal to participate will involve no penalty or loss of benefits or reduction in the level of care to which you are otherwise entitled to. You are also free to withdraw from the study at any point, even if you do agree to take part initially.

This study has been approved by the **Malawi University of Science and Technology** **Research Ethics Committee (MUSTREC)**. The study will be conducted according to the ethical guidelines and principles of the international Declaration of Helsinki, CIOMS Guidelines, Belmont Report, Guidelines for Good Clinical Practice and Ethical Guidelines for Research issued by the National Commission for Science and Technology

**What is this research study all about?**

- *This study is being conducted in commercial poultry farms in the city of Blantyre. The study is requiring participation of a poultry farmer with a total of 262 poultry farmers in Blantyre. The study is also requiring participation of at least a farm worker, 2 chickens and a poultry house for those farms to be sampled.*
- *We are actively investigating the extent of antibiotic resistance in bacteria isolated within poultry in Blantyre. This means we are studying how effective antibiotics are against these bacteria commonly found in chickens. Antibiotic resistance is a big problem because it makes it harder to treat infections, not only in animals but also in people who get sick from these bacteria. By understanding how much resistance there is, we can work on ways to protect both the health of the chickens and the people who eat poultry products. This study is important for creating strategies to prevent the spread of antibiotic-resistant bacteria and ensuring the safety of our food and health.*
- *We are collecting chicken droppings from the cloaca, human excreta from sewers or pit latrines, dust from chicken houses and hand swabs from the farm workers. We will analyse the collected samples at MUST microbiology laboratory. We are also asking farmers questions concerning their farm.*
- *To avoid bias, we are using randomisation in this study. We want to make sure we get a good mix of information from different types of poultry farms. So, we have divided the farms into two groups; broilers and layers. We are then randomly choosing farms from each group. We are also randomly collecting samples information from the farms. This division is called stratification. By using this process, we aim to get a diverse and representative set of samples and information, providing a more accurate picture of the overall situation in poultry farms.*

**Why do we invite you to participate?**

- *You are being invited to take part in this study of prevalence and antimicrobial resistance patterns of Escherichia coli and Klebsiella pneumoniae in poultry farms in Blantyre. The poultry industry is one of the growing food industries in Malawi hence antimicrobial resistance is of big concern.*

**What will your responsibilities be?**

- *If you agree to participate, and allow us to get information of your farm, you will be asked to sign this study consent form, and then answer a few questions concerning your chicken farm. The investigator will visit some of the farms once to collect faecal samples from chicken droppings, hand swabs from farm workers and human waste from sewers or pit latrines as well as dust from chicken houses.*

**Will you benefit from taking part in this research?**

- *The main benefit of participating in this study is that the study will identify risk factors for transmission of antimicrobial resistance infections and provide appropriate mitigation measures to the poultry farmers.* *This study will also help in fighting antimicrobial resistance by providing future researchers with information.*
- *For the farmers that will accept samples to be taken, if the chickens have resistant bacteria, it will be detected so that appropriate medications and other preventive measures can be implemented.*

**Are there in risks involved in your taking part in this research?**

- *The potential risk for participating is introduction of diseases on the farm. To avoid this, there will be minimal contact of the researchers with the chickens and all biosecurity on the farm will also be observed. This will be done to ensure that introduction of diseases on the farm is minimal.*

**If you do not agree to take part, what alternatives do you have?**

- *If you do not agree to take part in the study, you can still access veterinary services through the department of Animal Health and Livestock Production in the government sector.*

**Who will have access to your records?**

- *Your name and participation in the study will remain confidential and known only to the primary investigator. No identifying information such as name, address or any other information will be included in the study write-up or any related publications or presentations.*

**What will happen in the unlikely event of some form injury occurring as a direct result of your taking part in this research study?**

- *There will be no compensation for any form of injury occurring as a result of participating in this research study.*

**Will you be paid to take part in this study and are there any costs involved?**

- You will not have to pay for anything, if you do take part.
- *For those farmers that samples are collected from the farm, the amount of MK16,000 in form of cash will be paid.*

**Is there anything else that you should know or do?**

- You can phone Ms [MUONAOUZA DELEZA] at [0884654335] if you have any further queries about the study or encounter any problems during your participation in the study.
- You can contact the MUSTREC Administrator at 01 478 000 ext 8253 or [mustrec@must.ac.mw](mailto:mustrec@must.ac.mw) if you have any concerns or complaints that have not been adequately addressed by the researcher.
- You will receive a copy of this information and consent form for you to keep safe.
- **Declaration by participant**

By signing below, I …………………………………..…………. agree to take part in a research study entitled **(Prevalence and antimicrobial resistance patterns of *Escherichia coli* and *Klebsiella pneumoniae* in poultry farms in Blantyre**).

I declare that:

- I have read this information and consent form, or it was read to me, and it is written in a language in which I am fluent and with which I am comfortable.
- I have had a chance to ask questions and I am satisfied that all my questions have been answered.
- I understand that taking part in this study is **voluntary,** and I have not been pressurised to take part.
- I may choose to leave the study at any time and nothing bad will come of it – I will not be penalised or prejudiced in any way.
- I may be asked to leave the study before it has finished, if the study doctor or researcher feels it is in my best interests, or if I do not follow the study plan that we have agreed on.

Signed at (*place*) ......................…........…………….. on (*date*) …………....……….. 2024.

**Signature of participant**

- **Declaration by investigator/researcher**

I *(name)* ……………………………………………..……… declare that:

- I explained the information in this document to …………………………………...............
- I encouraged him/her to ask questions and took adequate time to answer them.
- I am satisfied that he/she adequately understands all aspects of the research, as discussed above.

Signed at (*place*) ......................…........…………….. on (*date*) …………....……….. 2024.

**Signature of investigator/researcher**

**For an illiterate participant**

A literate impartial witness must sign (if possible, this person should be selected by the participant and should have no connection to the research team). Participants who are illiterate should include their thumb-print as well.

**I have witnessed the accurate reading of the consent form to the potential participant, and the individual has had the opportunity to ask questions. I confirm that the individual has given consent freely.**

**Print name of witness:** ....................................................................

**Signature of witness:** .......................................................................

|  |
| --- |

**Thumb print of participant**

**Date: ______________________**

**Day/month/year**
