# Supplementary figures and images for: Antimicrobial susceptibility of Escherichia coli, Klebsiella pneumoniae, and Enterococcus species and the associated risk factors in poultry farms in Blantyre City: a wake-up call to the one health approach
Source: BMC Vet Res. 2025 Dec 23;22:73. doi: 10.1186/s12917-025-05189-7 (PMC12882281; doi:10.1186/s12917-025-05189-7)

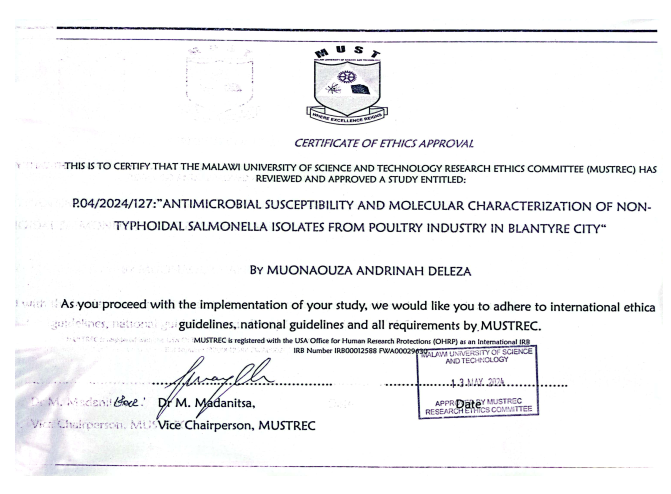

Supplement: Supplementary file 2 — Supplementary Material 2. [file 12917_2025_5189_MOESM2_ESM.png]
