# Supplementary material for: Antimicrobial susceptibility of Escherichia coli, Klebsiella pneumoniae, and Enterococcus species and the associated risk factors in poultry farms in Blantyre City: a wake-up call to the one health approach
Source: BMC Vet Res. 2025 Dec 23;22:73. doi: 10.1186/s12917-025-05189-7 (PMC12882281; doi:10.1186/s12917-025-05189-7)
Supplement: Supplementary file 3 — Supplementary Material 3. [file 12917_2025_5189_MOESM3_ESM.docx]

## **
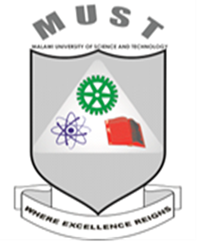
**

## **QUESTIONNAIRE**

1. FARM INFORMATION
2. Farm Name: ………………………………………………………………………
3. Location: …………………………………………………………………………..
4. Type of Farm: Broiler ……. Layer…….
5. Number of Chickens: ……………………………………………………………..
6. ANTIMICROBIAL USAGE
7. Have antibiotics been used on the farm for the 4 weeks?

Yes ………. No……….

1. If yes, please list the antibiotics used: …………………………………………..

………………………………………………………………………………………

1. BIOSECURITY MEASURES
2. Are there any biosecurity measures that are done on the farm?

Yes ………… No………

1. If yes, describe biosecurity measures implemented on the farm to prevent disease transmission: …………………………………………………………………………..

………………………………………………………………………………………

1. WORKER INTERACTION WITH CHICKENS
2. Do farmer workers have direct contact with the chickens?

Yes………. No………..

1. If yes, specify the nature of the contact (e.g., daily, weekly, monthly, rarely):

………………………………………………………………………………………..

1. PROTECTIVE MEASURES FOR WORKERS
2. Do farm workers use protective measures for prevention of disease transmission? Yes…………. No………………
3. If yes, what protective measures are in place for farm workers to prevent disease transmission? (e.g., gloves, gumboots): …………………………..

………………………………………………………………………………………….

1. ENVIRONMENTAL IMPACT
2. How is waste managed on your farm? (e.g. manure disposal, incineration: …………………………………………………………………………

……………………………………………………………………………………….

1. Do you believe there is a risk of contaminating the environment with the waste? Yes………….. No…………
2. If yes, please explain your concerns: ……………………………………………..

………………………………………………………………………………………..

1. KNOWLEDGE OF ANTIMICROBIAL RESISTANCE
2. Are you aware of the term ‘antimicrobial resistance’?

Yes …………… No……………

1. If yes, explain in your own words what it is: ……………………………………

………………………………………………………………………………………..

1. How are the risks of AMR managed at the farm? ……………………………….

………………………………………………………………………………………..

1. KNOWLEDGE OF ONE HEALTH
2. Are you aware of the term ‘One Health’ and its connection to the farm?

Yes ………… No………..

1. If yes, how do you think AMR might affect human health and the environment? ……………………………………………………………………………………………………………………………………………………………………………………
